# Supplementary material for: Among‐individual diet variation within a lake trout ecotype: Lack of stability of niche use
Source: Ecol Evol. 2021 Jan 19;11(3):1457–75. doi: 10.1002/ece3.7158 (PMC7863394; doi:10.1002/ece3.7158)
Supplement: Supplementary file 1 — Appendix S1 [file ECE3-11-1457-s001.docx]

**Appendix**

Table S1. Spatial and temporal information for the 79 Lake Trout classified as piscivorous ecotype from Great Bear Lake and analyzed for fatty acids. Sample sizes are in brackets.

|  | **Sample information** |
| --- | --- |
| **Group 1** (14) | Dease 2005 (1) Smith 2011 (1)  Dease 2010 (1)  McTavish 2009 (6)  McVicar 2008 (1)  Smith 2006 (4) |
| **Group 2** (16) | Dease 2010 (4) Smith 2006 (1)  Keith 2012 (2) Smith 2011 (5)  McTavish 2009 (1)  McTavish 2014 (2)  McVicar 2008 (1) |
| **Group 3** (21) | Dease 2005 (2) McVicar 2003 (2)  Dease 2010 (3) McVicar 2008 (2)  Keith 2012 (3) McVicar 2013 (3)  McTavish 2004 (1) Smith 2006 (1)  McTavish 2014 (2) Smith 2011 (2) |
| **Group 4** (28) | Dease 2005 (5) McTavish 2014 (1)  Dease 2010 (2) McVicar 2003 (2)  Keith 2002 (4) McVicar 2008 (5)  Keith 2003 (4) Smith 2006 (1)  MCTavish 2004 (2) Smith 2011 (1)  McTavish 2009 (1) |

Table S2. Bayesian clustering (i.e., STRUCTURE, Pritchard et al. 2000) results for piscivorous ecotype of lake trout from Great Bear Lake assessed using variation at 19 microsatellite markers. Shown are the mean log‐likelihood values (LnP[D]) for different hypothesized numbers of genetic populations (K) and the mean value of ΔK statistic of Evanno et al. (2005). Bold values represent the most likely number of genetic groups indicated by ΔK. Dashes = not applicable given that ΔK cannot be calculated for these values of K. For all STRUCTURE analyses, we employed an admixture model with the LOCPRIOR algorithm, correlated allelic frequencies, 100,000 burn-in and MCMC iterations and 10 iterations per K value were completed.

| K | Reps | Mean LnP(D) | Delta K |
| --- | --- | --- | --- |
| 1 | 10 | -10271.83 | — |
| 2 | 10 | **-10266.25** | **9.26** |
| 3 | 10 | -10572.68 | 0.03 |
| 4 | 10 | -10868.33 | 1.39 |
| 5 | 10 | -10739.53 | 0.45 |
| 6 | 10 | -10806.97 | 0.84 |
| 7 | 10 | -10678.37 | 0.66 |
| 8 | 10 | -10739.90 | 0.13 |
| 9 | 10 | -10862.98 | 1.09 |
| 10 | 10 | -10553.04 | — |

Table S3. Results of the discriminant analysis of principal components (DAPC, Jombart et al. 2010) implemented in the Adegenet package (Jombart et al. 2008) to determine the most likely number of genetic clusters (K) within the piscivorous Lake Trout ecotype from Great Bear Lake. The number of groups was identified using the find.clusters function (a sequential K-means clustering algorithm) and subsequent Bayesian Information Criterion (BIC), as suggested by Jombart et al. (2010). Stratified cross-validation carried out with the function *xvalDapc* was employed to determine the optimal number of PCs to retain in the analysis.

| **K** | **BIC** |
| --- | --- |
| 1 | 185.98 |
| 2 | 185.42 |
| 3 | 185.89 |
| 4 | 186.51 |
| 5 | 187.40 |
| 6 | 189.10 |
| 7 | 190.64 |
| 8 | 191.99 |
| 9 | 193.61 |
| 10 | 195.67 |

Table S4. Microsatellite loci used in this study and F_is_ values for each group per locus.

| **Locus** | **Group 1** | **Group 2** | **Group 3** | **Group 4** | **Giant** |
| --- | --- | --- | --- | --- | --- |
| **OtsG83b** | -0.021 | 0.017 | 0.046 | 0.090 | -0.013 |
| **Sco215** | 0.061 | 0.042 | -0.011 | -0.029 | 0.071 |
| **Smm17** | -0.433 | -0.069 | -0.038 | -0.304 | -0.012 |
| **Smm21** | -0.143 | -0.286 | -0.266 | 0.023 | 0.028 |
| **SnaMSU1** | 0.012 | -0.069 | -0.024 | -0.028 | -0.074 |
| **SnaMSU8** | -0.031 | 0.002 | 0.048 | 0.023 | 0.081 |
| **OMM1105** | 0.094 | -0.075 | -0.041 | -0.098 | -0.065 |
| **Smm22** | -0.014 | 0.055 | 0.082 | -0.088 | 0.137 |
| **SnaMSU13** | -0.105 | 0.053 | 0.136 | -0.073 | -0.049 |
| **SnaMSU5** | 0.088 | 0.065 | -0.032 | 0.039 | 0.159 |
| **Sco19** | -0.082 | 0.190 | 0.067 | -0.009 | 0.051 |
| **Sco202** | 0.107 | -0.166 | 0.047 | 0.115 | -0.080 |
| **SnaMSU10** | -0.108 | -0.030 | 0.086 | 0.096 | 0.203 |
| **SnaMSU12** | 0.122 | 0.069 | 0.123 | 0.201 | 0.072 |
| **SnaMSU6** | 0.008 | -0.090 | 0.002 | 0.007 | 0.207 |
| **Sal38** | -0.056 | 0.121 | -0.016 | -0.012 | 0.041 |
| **Sco200** | -0.015 | 0.098 | -0.096 | 0.041 | 0.244 |
| **SnaMSU11** | -0.060 | -0.108 | -0.012 | 0.083 | -0.011 |
| **SnaMSU3** | 0.059 | -0.019 | 0.065 | 0.012 | 0.085 |
| **Overall** | -0.027 | -0.011 | 0.009 | 0.005 | 0.057 |


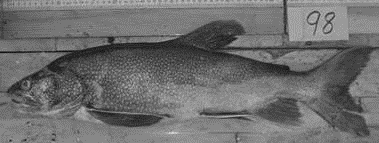

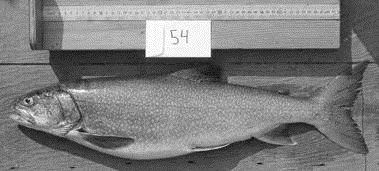

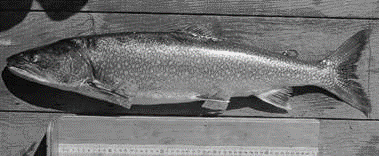

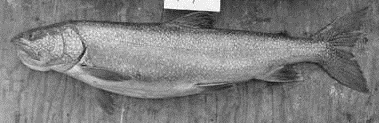


Morph 1

Morph 3

Morph 2

Morph 4

Fig. S1. The four shallow-water ecotypes of Lake Trout from Great Bear Lake identified in Chavarie et al. (2013, 2015, 2016a, 2016b): the generalist, the piscivore, the benthic-oriented, and the pelagic specialist, Morphs1-4, respectively.

**
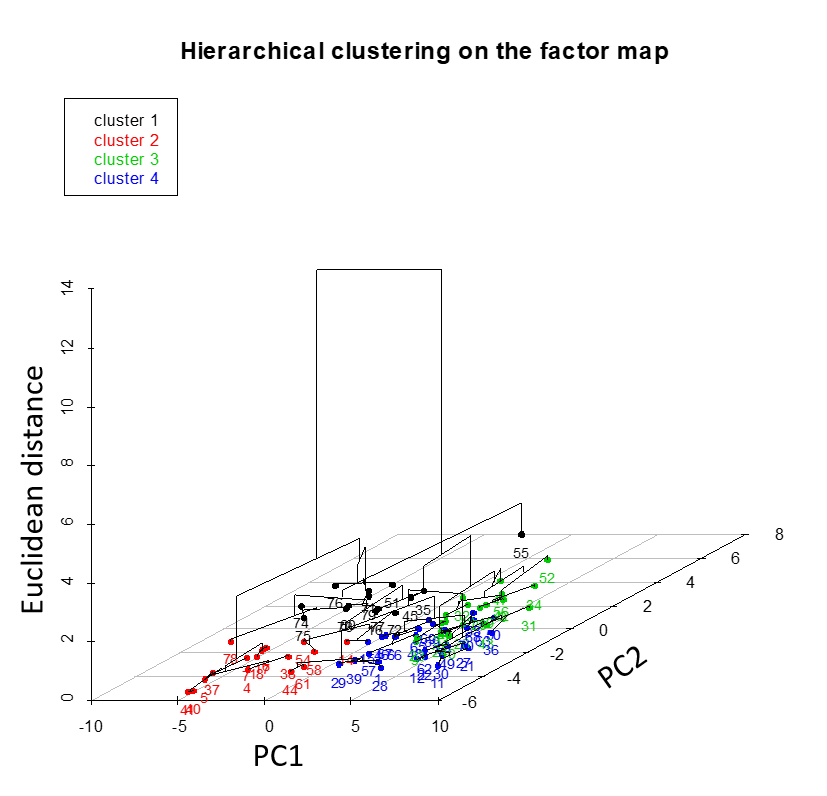
**

Fig. S2. Hierarchical clusters of Great Bear Lake Lake Trout fatty acids profiles overlaid on the first two principal component axes (PCA) using FactoMineR.


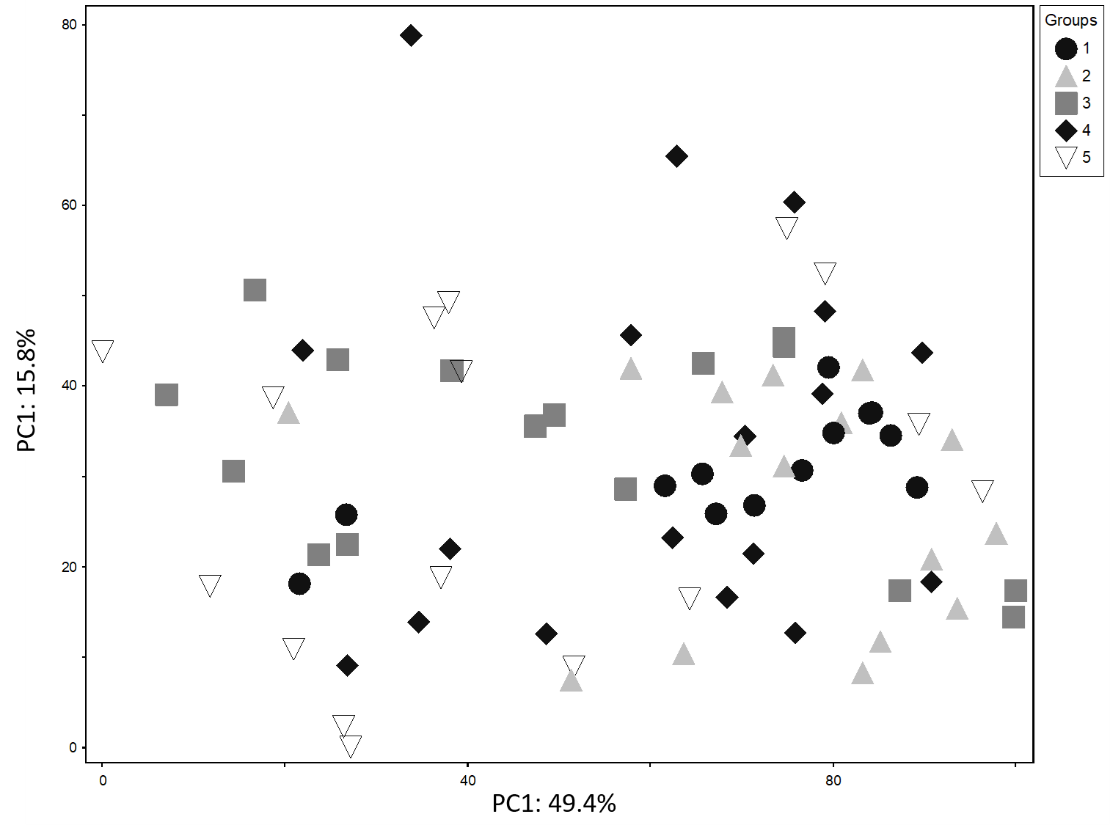


Fig. S3. Principal Component Analysis (PCA) of fatty acids of 79 Lake Trout classified as piscivorous ecotype from Great Bear Lake, based on the proportions of 41 fatty acids in dorsal muscle tissue. Spatial variations (5 arms; 1=Keith, 2=McVicar, 3=McTavish, 4=Dease, and 5=Smith) are represented, based on the fatty acids profile of each lake trout analyzed in this study.

Fig. S4. Depth of capture for four groups of piscivorous Lake Trout from Great Bear Lake (Groups identified by fatty acids profiles of individuals). Outliers are represented by a circle.


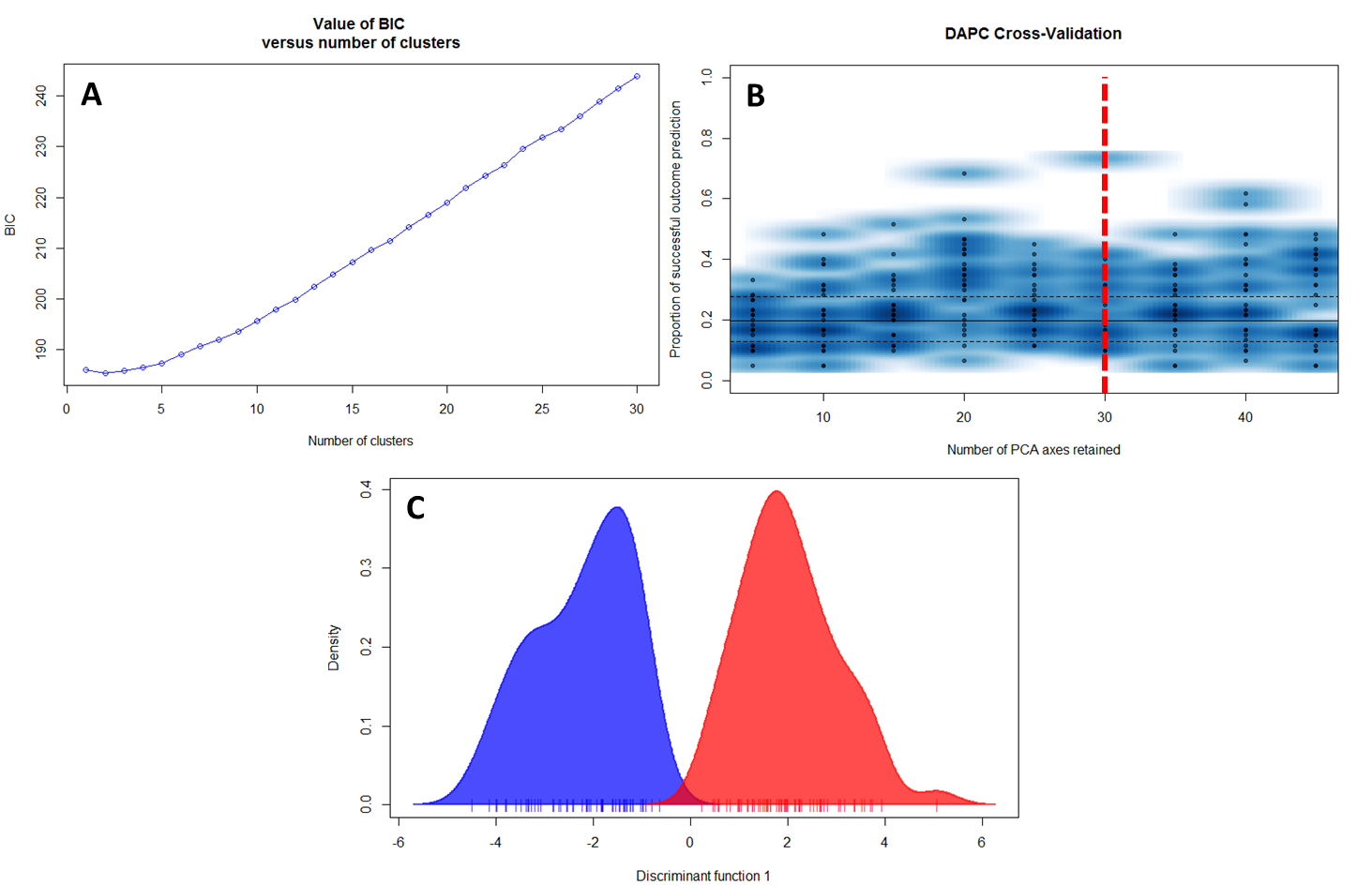


Fig. S5. Summary of the DAPC analysis. (A) Results of the cross-validation analysis used to determine the number of PCs to retain in the DAPC analysis. Cross-validation analysis determined the most appropriate number of PCs retained was 30. (B) Inference of the number of clusters in the DAPC performed on piscivorous Lake Trout from Great Bear Lake. The function find.clusters was run with a maximum number of clusters of 10 to identify the optimal number of clusters based on the BIC values. A K value of 2 (the lowest BIC value) represents the best summary of the data (most probable number of (K)). (C) The results of the discriminant function that shows that the two clusters are mostly non-overlapping.
